# Structural Characterization of *ABCB1*, the Gene Underlying the *d2* Dwarf Phenotype in Pearl Millet, *Cenchrus Americanus* (L.) Morrone

**DOI:** 10.1534/g3.118.200846

**Published:** 2018-06-17

**Authors:** Rajiv K. Parvathaneni, John J. Spiekerman, Hongye Zhou, Xiaomei Wu, Katrien M. Devos

**Affiliations:** * Institute of Plant Breeding, Genetics and Genomics,; † Dept. of Plant Biology, and; ‡ Dept. of Genetics, University of Georgia, Athens, GA 30602

**Keywords:** *d2* dwarfing gene, *ABCB1*, pearl millet, LTR-retrotransposon, heterologous transformation

## Abstract

Pearl millet is an important food crop in arid and semi-arid regions of South Asia and sub-Saharan Africa and is grown in Australia and the United States as a summer fodder crop. The *d2* dwarf germplasm has been widely used in the last half-century to develop high-performing pearl millet hybrids. We previously mapped the *d2* phenotype to a 1.6 cM region in linkage group (LG) 4 and identified the *ABCB1* gene as a candidate underlying the trait. Here, we report the sequence, structure and expression of *ABCB1* in tall (*D2D2*) and *d2* dwarf (*d2d2*) germplasm. The *ABCB1* allele in *d2* dwarfs differs from that in tall inbreds by the presence of two different high copy transposable elements, one in the coding region and the second located 664 bp upstream of the ATG start codon. These transposons were present in all *d2* dwarfs tested that were reported to be of independent origin and absent in the analyzed wild-type tall germplasm. We also compared the expression profile of this gene in different organs of multiple tall and *d2* dwarf inbreds, including the near-isogenic inbreds at the *d2* locus, Tift 23B (*D2D2*) and Tift 23DB (*d2d2*). Heterologous transformation of the tall (*Ca_ABCB1*) and the *d2* dwarf (*Ca_abcb1*) pearl millet alleles in the *Arabidopsis* double mutant *abcb1abcb19* showed that the pearl millet *D2* but not the *d2* allele complements the *Arabidopsis abcb1* mutation. Our studies also show the importance of the COOH-terminal 22 amino acids of the ABCB1 protein in either protein function or stability.

Modern agriculture has benefited tremendously from the improvements that breeders have made to crop plant architecture. In the 1960s, the widespread adoption of dwarf mutants in wheat (*Rht1*) and rice (*sd-1*) and the concomitant application of higher rates of fertilizer more than doubled yields (Pingali 2012). This was dubbed the ‘Green Revolution’ (reviewed by Hedden 2003). Height-reducing genes have also been extensively used in other cereals such as sorghum [*e.g. dw3* (Schertz *et al.* 1974)], barley [*e.g. denso* (Jia *et al.* 2009)], rye [*e.g. Ddw1* (Milach and Federizzi 2001)] and pearl millet [*e.g. d2* (Burton and Fortson 1966)]. Millets, of which pearl millet (*Cenchrus americanus* (L.) Morrone) is the most widely grown, are cultivated on ∼27 million hectares worldwide as a dual purpose food and fodder crop (Varshney *et al.* 2017). Pearl millet is also grown as a minor forage crop in the United States and Australia. In the developed world, pearl millet is cultivated exclusively as *d2* dwarf hybrids (Gulia *et al.* 2007). Tall pearl millet varieties are preferred in sub-Saharan Africa because the long stems are used for forage, fencing and roofing. In India, both tall and *d2* dwarf hybrids are grown depending on the region and season. In recent years, however, Indian farmers’ preference seems to have shifted toward tall hybrids as they have ‘good yield’ (grain and fodder), ‘good taste’, and a ‘good return value’ (Asare-Marfo *et al.* 2010). *d2* dwarfs yield less forage compared to tall pearl millet lines but the forage quality is higher due to a higher leaf to stem ratio (Johnson *et al.* 1968). Grain yield of *d2* dwarfs is lower in some backgrounds, but unaffected in others (Bidinger and Raju 1990; Rai and Rao 1991). Because *d2* dwarfs are more amenable to dense planting and machine harvesting, it is expected that the *d2* dwarfing trait will gain in importance as commercial agriculture in developing countries is moving toward mechanization.

Parvathaneni and colleagues identified a candidate gene for *d2* using a combination of high-density gene mapping, haplotype analysis of the *d2* region in tall and dwarf lines, and comparative genome analyses with rice and sorghum (Parvathaneni *et al.* 2013). The gene, *ABCB1*, encodes a P-glycoprotein (PGP) which facilitates cell to cell polar auxin transport. *ABCB1* belongs to the multidrug resistance (MDR)/P-glycoprotein (PGP) subfamily of the large superfamily ATP-binding cassette (ABC) transporters (reviewed in Dean *et al.* 2001; Sánchez-Fernández *et al.* 2001). Complete ABC transporter proteins contain four domains, including two transmembrane domains (TMDs) that bind specific substrates and two nuclear binding domains (NBDs) that hydrolyze ATP and use the resulting energy to transport the substrate. Half transporters contain one TMD and one NBD, and function either as homo- or heterodimers (Vasiliou *et al.* 2009). ABC transporters transport a wide range of compounds (reviewed in Kang *et al.* 2011). In plants, transported compounds include but are not limited to phytohormones, heavy metals, lipids, antibiotics, and glucosylated compounds. ABC proteins may also play a role in plant-pathogen interactions and in the modulation of ion channels (Rea 2007). There are eight ABC subfamilies, labeled ABCA to ABCH (Verrier *et al.* 2008). In plants, proteins of the ABCB (PGP) subfamily function in auxin transport, and are hypothesized to play an active role in the efflux of auxin from meristematic cells where the auxin concentration is very high (Blakeslee *et al.* 2005).

Reduced height as a result of loss-of-function mutations in *ABCB1* is well characterized in the panicoid grasses sorghum (*Sorghum bicolor – dw3* mutation) and maize (*Zea mays – br2* mutation) (Multani *et al.* 2003). An 881 bp tandem duplication in the fifth exon of *ABCB1* reduces internode length leading to a dwarf phenotype in sorghum (*dw3*). This phenotype is unstable because recombination between the direct repeats can give rise to dwarf-to-tall revertants. Interestingly, an unequal crossover event produced a stable *dw3* dwarf mutant that lacked the duplicated region and differed by the presence of several SNPs and one CG microsatellite in comparison to the *Dw3* allele. These mutations disrupt the reading frame and create a truncated protein that lacks 200 amino acids (Multani *et al.* 2003). The *dw3* mutation is widely deployed in sorghum breeding programs. Several recessive *Mu* insertional mutants of *ABCB1* causing dwarf phenotypes have been reported in maize (*br2*) (Multani *et al.* 2003). These insertion mutants have an extreme dwarf phenotype that cannot be exploited commercially. More recently, however, a *br2* allele has been identified that results in a 20% reduction in height and improves yield (Xing *et al.* 2015). This *br2* allele contains four synonymous SNPs and one non-synonymous SNP in the last exon of the *ABCB1* gene. The potential of mutating *ABCB1* genes to create agriculturally useful dwarfs is being explored using ethyl methanesulfonate (EMS) mutagenesis in the orphan crop tef (*Eragrostis tef*) to enhance lodging tolerance (Zhu *et al.* 2012).

In this manuscript, we describe the structural changes in *ABCB1* that led to its loss of function in the pearl millet *d2* dwarf. We validate *ABCB1* as underlying the *d2* mutation through expression analyses and transformation of the pearl millet functional and mutant alleles in an *Arabidopsis abcb1abcb19* dwarf background. Interestingly, modification of the 3′ end of the pearl millet *ABCB1* gene resulted in an intermediate phenotype in *Arabidopsis*. This mutation, when applied in monocots, might be useful for the generation of semi-dwarfs.

## Materials and Methods

### Plant material and DNA isolation

The pearl millet inbred lines used are listed with their genotype at the *D2* locus and their source in Table 1. Tift 23B and Tift 23DB are near-isogenic lines that differ for the presence of the *d2* allele. Inbred 81B is a selection from gamma-irradiation treated Tift 23DB (Supriya *et al.* 2011). The other *d2* lines are true-breeding segregants from tall landrace populations and have no known relationship (Appa Rao *et al.* 1986; ct hash, personal communication). Inbreds with allelic composition *D2D2* are tall, those with allelic composition *d2d2* are dwarf. The allele composition at the *D2* locus was unknown for the variety ‘Starr’. Burton and Devane (1951) reported ‘Starr’ to have morphological characteristics that fit the description of a *d2* dwarf. However, the ‘Starr millet’ (NSL 4716) obtained from the USDA National Plant Germplasm System (NPGS) was tall. Plants were grown in a growth chamber under a temperature of 26° and a 15/9 hr day/night cycle. DNA was isolated from leaves of three week old seedlings using either a CTAB extraction protocol (Murray and Thompson 1980) or a Qiagen DNA miniprep kit (Qiagen, Valencia, CA).

**Table 1:** Pearl millet inbred lines, their genotype at the *D2* locus, and source

Accession	Genotype at *D2* locus	Source
ICMP 451	*D2D2*	ICRISAT, Patancheru
Tift red	*D2D2*	Wayne Hanna, University of Georgia, Tifton
P-1449-2	*D2D2*	ICRISAT, Patancheru
Tift 23B	*D2D2*	Wayne Hanna, University of Georgia, Tifton
Tift 23DB	*d2d2*	Wayne Hanna, University of Georgia, Tifton
81B	*d2d2*	ICRISAT, Patancheru
pT 732B	*d2d2*	ICRISAT, Patancheru
IP 8008	*d2d2*	ICRISAT, Patancheru
IP 8058	*d2d2*	ICRISAT, Patancheru
IP 8112	*d2d2*	ICRISAT, Patancheru
IP 8157	*d2d2*	ICRISAT, Patancheru
IP 8208	*d2d2*	ICRISAT, Patancheru
IP 8227	*d2d2*	ICRISAT, Patancheru
IP 8288	*d2d2*	ICRISAT, Patancheru
IP 10399	*d2d2*	ICRISAT, Patancheru
Starr (NSL 4716)	Unknown	USDA, National Plant Germplasm System, Fort Collins, CO

The *Arabidopsis* single mutants *abcb1* (*atpgp1-2*; CS863226) and *abcb19* (*atmdr1-101*; SALK_033455) were obtained from the Arabidopsis Biological Resource Centre (ABRC). To generate an *abcb1abcb19* double mutant, the *abcb19* mutant (*atmdr1-101*) was crossed with the *abcb1* mutant (*atpgp1-2*). The *abcb1* mutant was used as pollen donor. F_1_ plants were selfed to generate F_2_ seed. F_2_ seed was germinated on half strength Murashige and Skoog (MS) (Murashige and Skoog 1962) agar plates and *abcb1abcb19* double mutants were identified phenotypically by their extreme dwarf stature. Presence of the T-DNA insertion in *ABCB1* was confirmed by PCR using primers 716F, LB2 and 716R, and in *ABCB19* using primers MDR1F, LB1a and MDR1R (Table 2).

**Table 2:** List of primers used and their annealing temperature

Primer	Sequence	Annealing temp. (°C)^1^
Ca_Sb07g023730_F1	5′-TACGCCTTCTACTTCCTCGTC-3′	
Ca_Sb07g023730_R5	5′-AGCAGCAGAAGACGGTGAAGTAG-3′
RB2_F1^1^	5′-ACTTGCCCCACTACAAGCAC-3′	61
D2UPF1	5′-CATGTTCCTTAATTCCTTTTTGC-3′	
D2UPR1	5′-ACTTGAGGAGGCACAAACTCATAC-3′	
D2UPRBF1^2^	5′-GAAGCCTTTGCTATCGTAGTGTG-3′	55
Ca_Sb07g023730_F10^3^	5′-GCAGGTTCTCCTTGATGCTC-3′	
Ca_Sb07g023730_R10^3^	5′-CTCGGAGGCACCTACTTCAC-3′	59
Ca_ABCB1_F20	5′-CATGACCTCAAGAGCCTGAA-3′	60
Ca_ABCB1_R20	5′-GAGCTTGATGATGAAGGAGTG-3′	
Ca_GAPDH_F1	5′-CTGTCGGTAAGGTTCTTCCTGAAT-3′	60
Ca_GAPDH_R1	5′-CTAACAGTGAGGTCAACAACTGACAC-3′	
Ca_Act_F1	5′-AGATCATGTTTGAGACCTTTGAATG-3′	60
Ca_Act_R1	5′-ATCACCAGAGTCCAGCACAATAC-3′	
D2T_F7	5′-CCGTTGAAGCTTCAGACGCCATTCCAAATCCCCATCTT-3′	72
D2T_R7	5′-CTGGTAAGGATCCGCTGCTGGTTGCTTCTTT-3′	
D2T_R16^4^	5′-GCGTTCCTTCCCAATGAGCTCTGC-3′	
D2T_F9^5^	5′-CCAAGATCTTCCGCATCATCGACCAC-3′	
D2T_Tail_R1	5′-CTGGTAAGGATCCCTAAGCATCATCTTCCTTAACCCTAGAACTT GAACCTGACGTCATACCAATCACTTGTGTATGCGTAAATCTTTG CAACTGCAGCATGCGCGCGTAG-3′	
D2T_F15	5′-CGTACTTGGGCATTTAACGCCCTGAC-3′	72
D2T_R10	5′-CGTAGAACCTCTCGATGAGCGACACGA-3′	
AtABCB1_F1	5′-CACCAACAACAACTCACGAAGC-3′	
AtABCB1_R1	5′-TAGACCCACAACATTCGAGACCCATC-3′	60
At_ActF1	5′-ATGAAGATTAAGGTCGTGGCA-3′	
At_ActR1	5′-GTGCACAAATGACAAAGGGGAA-3′	51
Br2F20	5′-CATGACCTCAAGAGCCTGAA-3′	
Br2R20	5′-GAGCTTGATGATGAAGGAGTG-3′	52
716F	5′-GAAGAGCCTAAGAAAGCAGA-3′	
716R	5′-GGTCAAATGGTGGCGAACTA-3′	
LB2^6^	5′-GCTTCCTATTATATCTTCCCAAATTACCAATACA-3′	53
MDR1F	5′-CATTTTATAATAACGCTGCGGACATAC-3′	
MDR1R	5′-CTTGAATCACACCAATGCAATCAAACACCTC-3′	
LB1a^7^	5′-TGGTTCACGTAGTGGGCCATCG-3′	60

1 Primer used in combination with Ca_Sb07g023730 R5.

2 Primer used in combination with D2UPR1.

3 From Parvathaneni *et al.* 2013.

4 Primer used in combination with D2T_F7.

5 Primer used in combination with D2T_R7.

6 Primer used in combination with 716R.

7 Primer used in combination with MDR1F.

### Isolation of a BAC clone carrying the ABCB1 allele from the dwarf inbred Tift 23DB

An available BAC library from the dwarf inbred line Tift 23DB, covering 5.6 genome equivalents and with an average insert size of ∼90 kb (Allouis *et al.* 2001), was screened for the presence of the *ABCB1* gene. The 159,100 clones that constitute the library were pooled per 384-well plate (single-plate pool) and DNA was extracted from each pool. DNA from 10 single-plate pools was combined into a super pool. PCR-based screening of super pools and single plate-pools was performed using the primer sets Ca_Sb07g023730_F10 and Ca_Sb07g023730_R10 (Table 2). PCR amplifications were performed in 20 µl reaction volumes containing 1X GoTaq Flexi PCR buffer (Promega), 1.5 mM MgCl_2_, 0.25 mM of each dNTP, 0.5 µM forward and reverse primers, 1 U of GoTaq DNA polymerase (Promega) and 25 ng of DNA template. Amplification conditions consisted of an initial denaturation step at 95° for 5 min followed by 34 cycles of 95° for 30 sec, 59° for 30 sec and 72° for 1 min, and a final extension of 72° for 5 min. Amplification products were run on 1% agarose gels. Once the plate address for a positive clone was identified, the clones in the corresponding 384-well plate were double-gridded on an Amersham HyBond N+ nylon membrane (GE Life Sciences) using a hand-held “colony-plaque lift” tool (V&P Scientific). Colonies were grown on the filters placed on LB medium with 25 µM chloramphenicol at 37° overnight. Lysis of the bacteria and denaturation of the DNA was performed according to established protocols (Sambrook *et al.* 2001). The 841 bp amplicon obtained in pearl millet with primer set Ca_Sb07g023730_F10/R10 was labeled using the Amersham Gene Images AlkPhos Direct labeling and Detection System. Probe labeling, colony hybridization and visualization of the hybridization sites were performed using the manufacturer’s recommendations for the Amersham Gene Images AlkPhos Direct labeling and Detection System with the following modifications. To decrease non-specific hybridization, membranes were pre-hybridized with hybridization buffer at 65° for 45 min. The hybridization and 2X primary wash were also performed at 65°. Fluorescent signals were recorded on a high performance chemiluminescence film (Amersham Hyperfilm ECL).

### Isolation of a fosmid clone carrying the ABCB1 allele from the tall inbred ICMP 451

To isolate the *ABCB1* allele from a tall inbred, a fosmid library was constructed of ICMP 451. High molecular weight DNA was isolated from nuclei using a modified protocol from Peterson and colleagues (Peterson *et al.* 2000). To shear the DNA to an average size of 35 kb, 10 µg of high quality nuclear DNA (50 to 100 ng/µl in 10 mM TE) was added to 1.5 volumes of AP3 buffer provided in the DNeasy plant mini kit (Qiagen), and passed through a DNeasy mini spin column (Qiagen). The manufacturer’s protocol was then followed to recover the nuclear DNA. The DNA was separated for 16 hr on a 1% low melting point agarose gel in 0.5X TBE buffer by field inversion gel electrophoresis (FIGE) (BIO-RAD FIGE Mapper Electrophoresis System) with forward pulses of 180V, reverse pulses of 120V, and a linear switch time ramp of 0.1-2.0 sec. A 5-50 kb pulsed field molecular weight ladder (Lambda DNA monocot mix, NEB) and a 42 kb control DNA sample from the Copy Control Library Production Kit (Epicentre Biotechnologies, Madison, USA) were used as standards. Fragments in the size range 30-43 kb were eluted from the gel using the components of the Copy Control Fosmid Library Production Kit (Epicentre Biotechnologies, Madison, USA) and used to construct a library following the manufacturer’s recommendations. Fosmids were plated at a density of ∼800 clones per plate. Colonies from a single plate were combined using LB broth to constitute a single pool and stored as a 20% glycerol stock. Ten µl of bacterial culture from eight pools were combined to constitute super pools for a total of 24 super pools. The same primer sets and conditions as used in the BAC library screening were used to screen the fosmid library using 0.5 µl of super pool culture as template. Once the address of the pool was known, the culture from the pool was titrated and plated to give ∼100 colonies per plate. Twelve sub-pools, each consisting of ∼100 colonies, were screened by PCR for each positive pool. The positive sub-pools were plated again and individual colonies were screened by PCR to identify the fosmid that contained the *ABCB1* gene.

### Sequencing of the BAC and fosmid clones

Plasmid DNA was isolated from the *ABCB1*-positive fosmid (fosmid-19) and BAC (156A12) clones using the Qiagen Large Construct Kit (Qiagen). DNA of fosmid-19 and BAC 156A12 was fragmented to an average size of 900 bp by nebulization and cleaned using AMpure beads (Agencourt Bioscience, Beverly, MA). Fragments were then end-repaired and ligated to adapters at the Georgia Genomics and Bioinformatics Core (GGBC), UGA, using in-house protocols. Libraries were paired-end sequenced using the Roche 454 GS FLX sequencing platform with Titanium chemistry. BAC 156A12 was sequenced to a depth of 67X and fosmid-19 to a depth of 23X. Summary statistics for the sequenced BAC and fosmid are provided in Table S1. Sequences were assembled *de novo* with MIRA v. 3.2.0 (Li *et al.* 2008) using the ‘normal’ mode for the assembly of BAC 156A12 and ‘accurate’ settings for assembly of the fosmid-19 clone. Contigs were ordered based on synteny information obtained by conducting a BLASTN search using the pearl millet contigs against the masked genome sequences of *Setaria italica* (assembly v2.2) and *Sorghum bicolor* (assembly v3.1.1) available from Phytozome (phytozome.jgi.doe.gov). Contigs longer than 2 kb without gene homologs were placed at the end.

### Classification of the transposable elements present in the d2 allele

Sequences of the transposable elements were translated in six frames and compared to the hidden Markov model (HMM) profiles deposited in GyDB 2.0 (Llorens *et al.* 2011) to find the reverse transcriptase (RT) domains. The RT domains were used in an hmmsearch (HMMER version 3.0 package; www.hmmer.org) to identify the TE superfamily and clade to which the elements belonged. The closest transposable elements were identified by BLASTN analysis of the DNA sequence of the elements to the MIPS repeat element database (Nussbaumer *et al.* 2013) and Repbase (Jurka *et al.* 2005). Because the assembled 5′ and 3′ LTR regions were identical, we calculated what the insertion time would have been if the two LTRs varied by 1 bp. The actual insertion time falls between that time point and the first discovery of the dwarf mutant in the 20^th^ century. To calculate the earliest date of insertion, we used a k-value of (1/921)/site for ‘Juriah’ and (1/883)/site for ‘Parel’ (k = substitution rate/site), an r-value of 1.3 × 10^−8^ substitutions/site/year (Ma and Bennetzen 2004) and the formula t (divergence time) = k/2r (Sanmiguel *et al.* 1998).

### Prevalence and location of ‘Juriah’ and ‘Parel’ LTR-retrotransposons in the pearl millet genome

Repeatmasker version 4.0.5 was used to determine the percentage of Juriah and Parel elements in the pearl millet genome (Varshney *et al.* 2017). The ‘Juriah’ and ‘Parel’ sequences as annotated in BAC 156A12 (Genbank accession MH059799) were used as customized library for input in RepeatMasker. The parameter settings of Repeatmasker were ‘–e wublast –nolow’.

### PCR testing for the presence of the ‘Juriah’ and ‘Parel’ transposons in tall and dwarf pearl millet accessions

The primer set RB2F1/Ca_Sb07g023730R5 (Table 2) spans the 3′ boundary of *ABCB1* and the ‘Juriah’ LTR. This primer set was used to test tall and *d2* dwarf inbreds for the presence of the ‘Juriah’ element. Some plants were also tested with primer set Ca_Sb07g023730 F1/R5, which flanks the ‘Juriah’ transposable element. Similarly, primer set d2UPRBF1/D2UPR1 spans the boundary of *ABCB1* and ‘Parel’, while primers D2UPF1 and D2UPR1 flank ‘Parel’. PCR amplifications were performed in 20 µl reaction volumes containing 1X GoTaq Flexi PCR Buffer (Promega), 1.5 mM MgCl_2_, 0.25 mM of each dNTP, 0.5 µM forward and reverse primers, 1 U of GoTaq DNA polymerase (Promega) and 25 ng of DNA template. Amplification conditions consisted of an initial denaturation step at 95° for 5 min followed by 34 cycles of 95° for 30 sec, 61° (for ‘Juriah’) or 55° (for ‘Parel’) for 30 sec and 72° for 1 min, and a final extension at 72° for 5 min. Amplification products were run on 1% agarose gels.

### Expression analyses

For expression analyses, three tall inbred lines (ICMP 451, P-1449-2 and Tift 23B; three plants per genotype) and three dwarf inbred lines (81B, pT 732B and Tift 23DB; three plants per genotype) were grown in 6 inch pots (for analysis at the seedling stage) or 12 inch pots (for analysis of adult tissues) in the greenhouse under 14 hr day lengths and a day/night temperature of approximately 27°/21°. When seedlings reached the five-leaf stage (Figure S1A), the oldest leaf and the stem were collected for expression analyses. The panicle, top node, top internode and root were collected when 50% of the stigma had emerged (Figures S1B and S1C). Samples were flash frozen in liquid nitrogen and stored at -80° until the time of RNA extraction. RNA was extracted using a standard protocol using TRIzol reagent (Chomczynski and Sacchi 1987). The RNA quality was checked on a 1% agarose gel. Up to 5 µg of RNA was treated with DNase using the Ambion TURBO DNA-*free* kit after which the RNA was quantified using a nanodrop (NanoDrop technologies). A total of 800 ng (seedling tissues) or 500 ng (adult tissues) of DNase treated total RNA was used to conduct cDNA synthesis with the SuperScript III Reverse Transcriptase system (Life Technologies) using an OligodT_20_ primer according to the manufacturer’s recommendations. At least three biological replicates were analyzed for each sample.

Quantitative RT-PCR was conducted as detailed in Dash and Malladi (2012). In brief, the Veriquest SYBR Green qPCR master mix (Affymetrix, Santa Clara, CA) was used with the Stratagene Mx3005P real-time PCR system (Agilent Technologies, Santa Clara, CA). PCR conditions were as follows: 50° for 2 min, 95° for 10 min, and 40 cycles of 95° for 30 s and 60° for 1 min. A melting curve analysis was performed at the end of the cycles to check for single peaks, which indicate amplification of a single fragment. The primers Ca_ABCB1_F20 and Ca_ABCB1_R20 (Table 2) were designed to quantify expression of the pearl millet *ABCB1* gene. The pearl millet *ACTIN* gene (amplified with primers Ca_Act_F1/R1; Table 2) and glyceraldehyde 3-phophate dehydrogenase (*GAPDH*) gene (amplified with primers Ca_GAPDH_F1/R1; Table 2) were used as references to correct for variation in the amount of input cDNA. One complete set of replicates was analyzed per PCR run. The primer efficiency (PE) was calculated using the program LinRegPCR for each run using the amplification plots (Ruijter *et al.* 2009). The relative quantity (RQ) of each amplicon was calculated as 1/(PE*Ct) with Ct the number of PCR cycles required to cross a fluorescence threshold of 0.1. The normalized relative quantity (NRQ) of the gene-specific amplicons was calculated by dividing their relative quantity (RQ) by the normalization factor (geometric mean of RQ for *ACTIN* and *GAPDH*). Transcript levels of *ABCB1* in the different genotypes were determined relative to the average NRQ value for the same tissue and developmental stage in Tift 23B. T-tests on the log_2_ (NRQ) values were used to identify statistically significant differences in transcript levels.

### Generation of the constructs for transformation

Five constructs, referred to as D2, d2, D2_AtTail, d2_AtTail and AtCDNA were tested for their ability to rescue the phenotype of an *Arabidopsis abcb1abcb19* double mutant.

To amplify the full-length *ABCB1* coding region from the tall inbred ICMP 451 (D2 construct), primers were designed 162 bp upstream of the start codon in the 5′UTR (primer D2T_F7) and 108 bp downstream of the stop codon in the 3′UTR region (primer D2T_R7) of pearl millet *ABCB1*. Primers D2T_F7 and D2T_R7 (Table 2) were designed to carry a *Hin*dIII and *Bam*HI restriction site, respectively. PCR amplification was conducted using the high-fidelity DNA polymerase Q5 (NEB) in a 25 µl reacting volume consisting of 1X reaction buffer, 1X enhancer buffer, 0.2 mM of each dNTP, 0.5 µM forward and reverse primers, 0.5 U of Q5 DNA polymerase (NEB), and 1 µl (50 ng/µl total RNA) of ICMP 451 cDNA. PCR reactions were assembled on ice, and then placed in a PCR block preheated to 98^°^. PCR conditions were as follows: an initial denaturation at 98^°^ for 30 sec; 30 cycles of 98^°^ for 10 sec and 72^°^ for 3 min; and a final extension at 72^°^ for 8 min.

To generate a non-functional pearl millet *ABCB1* allele comparable to the *ABCB1* allele present in the dwarf line Tift 23DB (d2 construct), three overlapping fragments were PCR-amplified and subsequently joined by a further round of PCR (Figure S2). Primer pair D2T_F7/D2T_R16 (Table 2) was used on DNA of Tift 23DB BAC clone 156A12 to generate ‘fragment 1’ which captured 162 bp of the 5′UTR, the region of *ABCB1* exon 1 upstream of the ‘Juriah’ TE and 815 bp of the 5′ LTR of ‘Juriah’. ‘Fragment 2’ was amplified from BAC 156A12 with primer set D2T_F15/D2T_R10 (Table 2) and contained 1003 bp of the 3′ LTR of the ‘Juriah’ TE and most of the exon 1 region downstream of ‘Juriah’ (Figure S2A). Fragment 1 and fragment 2 overlapped by 405 bp due to sequence homology between the 5′ and 3′ LTRs. Primer set D2T_F9/D2T_R7 (Table 2) was used on the D2 construct to capture the remainder of the coding region of *ABCB1* (‘fragment 3′) (Figure S2B). Fragment 2 and fragment 3 overlapped by 240 bp in exon 1. The three PCR fragments were cleaned using the Qiagen PCR purification kit (Qiagen, Valencia, CA). PCR with primer set D2T_F7/D2T_R7 and a mix of fragments 1, 2 and 3 as template generated an amplicon that was essentially identical to the *d2* coding region except that a ‘Juriah’ solo LTR was present in exon 1 instead of the full length LTR retrotransposon (Figure S2C). All PCR amplifications were conducted using the Q5 high-fidelity polymerase (NEB).

Primers AtABCB1_F1 and AtABCB1_R1 (Table 2) were used to amplify the full length coding region of *ABCB1* from *Arabidopsis Col-0* cDNA (construct AtCDNA). *Bam*HI and *Avr*II restriction sites were added to the primers for cloning. The tail constructs were made using the generated D2 and d2 constructs as template. Amplification was done using primer D2T_F7 as forward primer and D2T_Tail_R1 as reverse primer (Table 2). Primer D2T_Tail_R1 incorporated the last 78 bp of the *ABCB1* coding region in *Arabidopsis*.

The amplified coding sequences were purified using the Qiagen PCR purification kit, double digested with *Hin*dIII/*Avr*II and *Bam*HI, and cloned in an engineered T-DNA pCambia vector, referred to as pCambia1300_OX, which conferred kanamycin resistance in *E. coli* and hygromycin resistance in plants. pCambia1300_OX was developed in-house by replacing the multiple cloning site (MCS) of the pCambia1300 vector (Hajdukiewicz *et al.* 1994) with the ‘CaMV 35S promoter:multiple cloning site:nopaline synthase (NOS terminator)’ from PCCN3_S_OX (construct developed and provided by Wolfgang Lukowitz, UGA). The correctness of the pCambia1300_OX construct and of the inserts was verified by Sanger sequencing. Approximately 100 ng of each plasmid was then transformed into *Agrobacterium tumefaciens* strain GV3101 (Koncz and Schell 1986).

### Transformation studies in the Arabidopsis abcb1abcb19 mutant

The *A. tumefaciens* strain GV3101 containing the pCambia1300_OX vector with different inserts was used to transform the *Arabidopsis abcb1abcb19* double mutant using the floral dip method (Clough and Bent 1998). T1 seeds were collected and germinated on 1/2 strength MS agar plates with 25 µg/ml hygromycin. The plates were placed at 4° for 3 days and then transferred to a growth chamber under continuous light. After 13 days in the growth chamber, the surviving plants were transplanted into small pots, grown under long days (16 hr day length) and selfed to generate T2 lines.

### Phenotypic characterization of transformants

Two to 14 plants each from at least three independent transgenic T2 lines for each of five constructs (AtCDNA, D2, d2, D2_AtTail and d2_AtTail) were grown in a growth chamber under short-day conditions (8 hr light) together with wild-type (Columbia ecotype), *abcb1* mutant, *abcb19* mutant, and *abcb1abcb19* double mutant plants. Phenotypic measurements included rosette width (measured at its widest point at the onset of floral initiation), leaf length and width (measured on the three longest, still-green leaves at the onset of floral initiation), time of inflorescence emergence (when the inflorescence extended 1 cm above the rosette surface) and inflorescence height at maturity. For transgenic lines with a tall phenotype in the T1 generation, the dwarf:tall ratio was recorded in the T2 generation. Significant differences between trait measurements (adjusted P-values) were calculated using one-way ANOVAs followed by *post-hoc* Tukey’s honest significant difference (HSD) tests with multiple comparisons across means.

### Expression analysis of the transformants

Expression of the introduced genes was analyzed in a single representative T_2_ plant for each independent transgenic line via semi-quantitative (semi-q) RT-PCR. RNA was isolated from leaf tissue using a Qiagen RNeasy Plant Mini Kit, and cDNA was synthesized using oligo(dT) primers and a Thermo Scientific RevertAid First Strand cDNA Synthesis Kit. Semi-qRT-PCR was conducted in 25 µl reaction volumes containing 1X GoTaq Flexi PCR buffer (Promega), 1.5 mM MgCl_2_, 0.25 mM of each dNTP, 0.5 µM forward and reverse primers, 1 U of GoTaq DNA polymerase (Promega) and 1 µl of each cDNA reaction mix. PCR conditions were as follows: initial denaturation at 95° for 2 min, followed by 25 cycles (for D2, d2, D2_AtTail and d2_AtTail construct expression) or 26 cycles (for AtCDNA construct and *ACTIN* expression) of denaturation at 95° for 30 sec, annealing at 52° for 30 sec, and extension at 72° for 1 min with a final extension at 72° for 5 min. Primers used for semi-qRT-PCR were Br2F20/Br2R20 for expression of the D2, d2, D2_AtTail and d2_AtTail constructs, ABCB1-F1/ABCB1-R1 for expression of the AtCDNA construct, and At_ActF1/At_ActR1 for expression of the *Arabidopsis ACTIN 8* gene (*ACT8*) as the control (Table 2). Amplicons were run on a 1.5% agarose gel, and band intensities were quantified using the ImageJ software (Abràmoff *et al.* 2004).

### Data availability

The *Arabidopsis abcb1abcb19* double mutant generated as part of this project, and the plasmids used in this study are available upon request. The sequences of fosmid-19 comprising the *D2* allele and of BAC 156A12 comprising the *d2* allele are available under Genbank accession numbers MH059798 and MH059799, respectively. All data necessary to confirm the results and support conclusions of the article are present in the paper. Supplemental material available at Figshare: https://doi.org/10.25387/g3.8185955.

## Results

### The pearl millet ABCB1 gene

The total coverage of the fosmid library generated from the pearl millet tall inbred ICMP 451 was estimated at 2.2 genome equivalents. A clone carrying *ABCB1*, fosmid-19, was sequenced to a depth of 23X and assembled into one large contig (C1; 31,571 bp) and one smaller contig (C2; 6952 bp) (Genbank accession number MH059798). The summary statistics for the fosmid-19 assembly, including raw read number, read length and number of contigs are provided in Table S1. The complete *ABCB1* gene was comprised within contig C1. The genes identified in contigs C1 and C2 based on their homology to annotated sorghum and *Setaria* genes are listed in Table 3.

**Table 3:** Comparative presence of genes in fosmid-19 and BAC 156A12, sorghum chromosome 7 and *S. italica* chromosome VI

Sorghum^1^	Setaria^1^	Fosmid-19	BAC 156A12
Sobic.007G163100	Seita.6G253900	Fosmid – C2	—
Sobic.007G163200	—	—	—
Sobic.007G163300	—	—	—
Sobic.007G163400	Seita.6G253800	Fosmid – C2	—
Sobic.007G163433	—	—	—
Sobic.007G163466	—	—	—
Sobic.007G163500	[Seita.6G255200]^2^	—	—
Sobic.007G163600	Seita.6G253700	Fosmid – C1	—
Sobic.007G163700	Seita.6G253600	Fosmid – C1	156A12 – C2
Sobic.007G163800 (*ABCB1*)	Seita.6G253500 (*ABCB1*)	Fosmid – C1	156A12 – C2/C6
Sobic.007G163901	—	—	—
Sobic.007G164000	[Seita.7G250000]^2^	Fosmid – C1	156A12 – C5/C1

1 Orthology with sorghum and *Setaria* genes was determined by reciprocal BLASTN analysis of the coding region against the whole genome sequence.

2 Genes within square brackets are orthologs located in non-colinear positions in *Setaria*.

**Figure 1:**
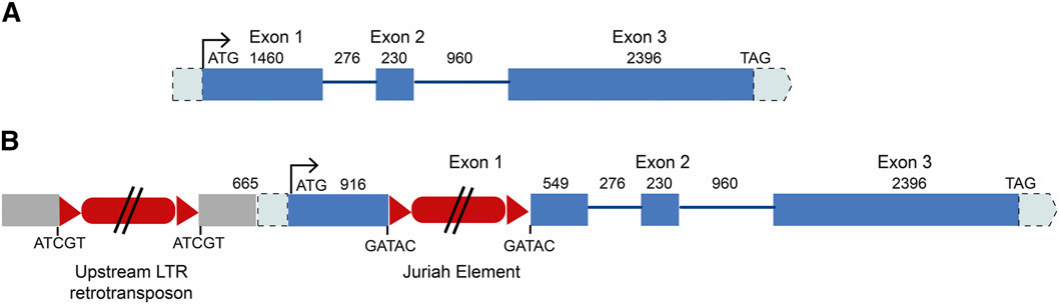
Structure of pearl millet *ABCB1* alleles. A) *Ca_ABCB1* isolated from the tall inbred line ICMP 451, and B) *Ca_abcb1* isolated from the dwarf inbred line Tift 23DB. The sizes of the 5′UTR and 3′UTR regions are unknown. Exons are represented by blue boxes, introns as lines and LTR-retrotransposons as red boxes. Red triangles indicate the long terminal repeats (LTRs). The ‘ATCGT’ and ‘GATAC’ sequences are the 5-bp direct repeats flanking the LTR-retrotransposons. The figure is not drawn to scale.

Sequence analysis of the *ABCB1* gene from ICMP 451, hereafter referred to as *Ca*_*ABCB1*, revealed that it contained three exons and two introns (Figure 1A), the same as *ABCB1* in foxtail millet (GenBank acc. XM_004974196). In contrast, the maize and sorghum *ABCB1* orthologs, *Zm*_*ABCB1* (*Br2*) (GenBank acc. AY366085) and *Sb_ABCB1* (*dw3*) (GenBank acc. AY372819), respectively, consisted of five exons and four introns. A neighbor joining tree of ABCB1 and ABCB19, the closest homolog to ABCB1, proteins showed that pearl millet ABCB1 falls in its expected phylogenetic position within the ABCB1 clade (Figure S3), confirming the identity of *Ca_ABCB1*. Pearl millet ABCB1 has 96% homology with foxtail millet ABCB1, 89% with sorghum ABCB1 and 88% with maize ABCB1 at the amino acid level with a query coverage of 99%.

### The ABCB1 allele in the d2 dwarf Tift 23DB

BAC clone 156A12, derived from the dwarf variety Tift 23DB, was sequenced to a depth of 67X and assembled into 14 contigs (Genbank accession number MH059799). Contig size varied from 457 bp to 47,002 bp. Four contigs (C1, C2, C5 and C6) carried genes. *ABCB1* was distributed across contigs C6 and C2 (Table 3), which had total lengths of 2586 bp and 23,307 bp, respectively. The lack of assembly across the entire length of the *ABCB1* gene was caused by the presence of a LTR retrotransposon in exon 1. The 5′ end of the 5′ LTR was located on contig C6, and the 3′ region of the 5′ LTR, the internal region of the transposon and the 3′ LTR were present on contig C2. With the exception of a single base length variation in a poly(G) tract which was likely a 454 sequencing error, the available (921 bp out of 1408 bp) 5′ LTR sequence was identical to the 3′ LTR sequence. The 5-bp host target site duplication was GATAC. The LTR retrotransposon had 94% homology along its full length to an annotated ‘Juriah’ element in *Cenchrus americanus* BAC 311G2 (Genbank accession AF488414). The ‘Juriah’ element belongs to the ‘tat’ clade of the ‘gypsy’ superfamily. An analysis of the presence of ‘Juriah’ in the pearl millet genome (http://gigadb.org/dataset/100192) showed that this element is high copy and represents ∼7% of the pearl millet genome.

In addition to the differential presence of the ‘Juriah’ LTR retrotransposon in exon 1, the coding regions of the *ABCB1* alleles isolated from ICMP 451 and Tift 23DB varied by the presence of a synonymous SNP in exon 1. The 5 kb upstream regions varied by 10 SNPs, two indels (one of 1 bp and one of 13 bp), the length (1 unit difference) of a (CT) simple sequence repeat (SSR), the length of two (T) homopolymers and the insertion of a second LTR retrotransposon (Figure 1). This TE was located 664 bp upstream of the ATG start codon and, hence, was presumably located in the *ABCB1* promotor. The TE was spread over contigs C6, C11, C3 and C5. Manual investigation showed that contigs C6 and C11, and C11 and C3 overlapped so that the 3′ LTR, the internal region of the transposon and the 3′ end of the 5′ LTR were located on the joined contig C6rev-C11-C3rev, and the 5′ end of the 5′ LTR was located on contig C5. The host target site duplication was ATCGT. The reverse transcriptase domain of this element had the best hit to the ‘del’ clade of gypsy elements. As this element had limited homology to known LTR retrotransposons, it was classified as a new element and named ‘Parel’ (Table S2). ‘Parel’ made up ∼2% of the pearl millet genome.

The BAC 156A12 contigs were ordered (C1rev-C5-C3-C11rev-C6-C2rev) based on the structure of the ‘Juriah’ and ‘Parel’ transposable elements, the structure of the *ABCB1* gene and synteny with *Setaria italica* (foxtail millet) and *Sorghum bicolor* (sorghum) (Table 3). These six contigs plus two contigs larger than 2 kb that were unordered and not oriented (C4, 13,851 bp and C12, 2224 bp) totaled 106,305 bp.

### Presence of the ‘Juriah’ element in dwarf and tall pearl millet inbreds

**Figure 2:**
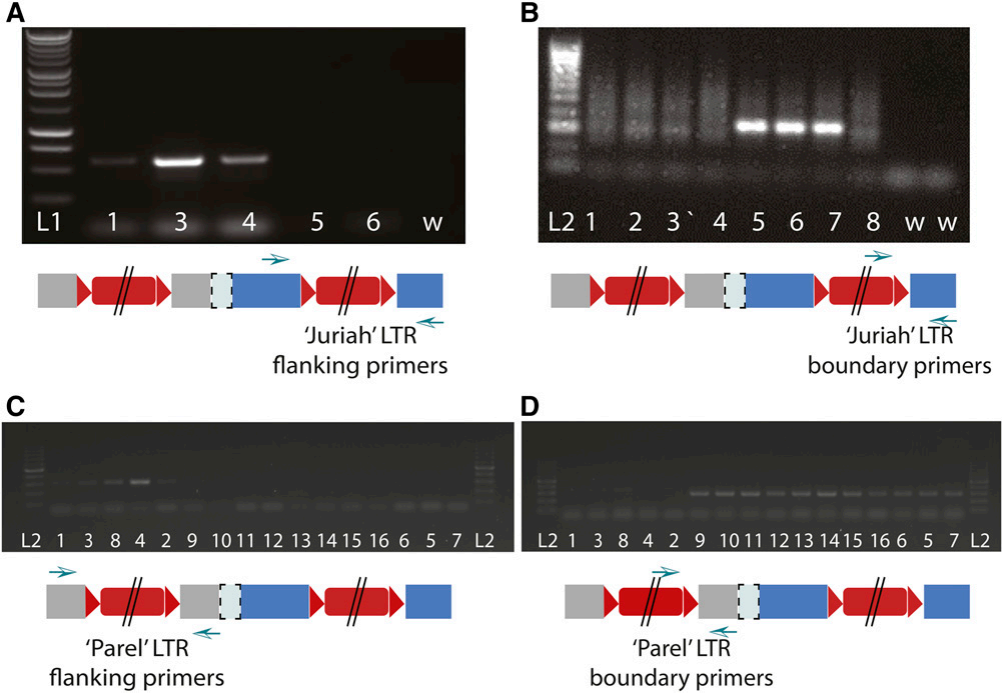
PCR amplification of diverse pearl millet genotypes using A) *ABCB1*-specific primers flanking the ‘Juriah’ LTR-TE; and B) a forward primer specific to the ‘Juriah’ LTR-TE in combination with an *ABCB1*-specific reverse primer. C) *ABCB1* promoter-specific primers flanking the ‘Parel’ LTR-TE; D) a forward primer specific to the ‘Parel’ LTR-TE in combination with an *ABCB1* promoter-specific reverse primer; L1: 1 kb ladder; L2: 100 bp ladder; w: Water controls; 1. ICMP 451 (*D2D2*); 2. Tift 23B (*D2D2*); 3. Tift red (*D2D2*); 4. P-1449-2 (*D2D2*); 5. Tift 23DB (*d2d2*); 6. 81B (*d2d2*); 7. pT 732B (*d2d2*); 8. ‘Starr’ millet; 9. IP 8288; 10. IP 8008; 11. IP 8227; 12. IP 8058; 13. IP 8112; 14. IP 8208; 15. IP 8157; 16. IP 10339.

Five tall inbreds (ICMP 451, Tift red, Tift 23B, P-1449-2 and ‘Starr’) and 11 *d2* dwarf inbreds (Tift 23DB, 81B, pT 732B, IP 8227, IP 8208, IP 8288, IP 8157, IP 8112, IP 8008, IP 8058 and IP 10399) were tested for the presence of the ‘Juriah’ LTR-retrotransposon using a primer set that spanned the 3′ boundary between *ABCB1* and the ‘Juriah’ transposon. A subset of the plants were also tested with primer set Ca_Sb07g023730 F1/R5, which flanked the ‘Juriah’ transposable element. The PCR results demonstrated that the ‘Juriah’ element was absent from the five tall inbreds tested and present in all *d2* dwarf inbred lines tested (Figures 2A and 2B). The same accessions were similarly tested for the presence of the ‘Parel’ element using both a primer set that flanked the *ABCB1* – TE junction and a primer set that flanked the TE. The ‘Parel’ element was absent from all tall inbred lines and present in the *d2* dwarf inbred lines (Figures 2C and 2D).

### Expression analyses of Ca_ABCB1 and Ca_abcb1

**Figure 3:**
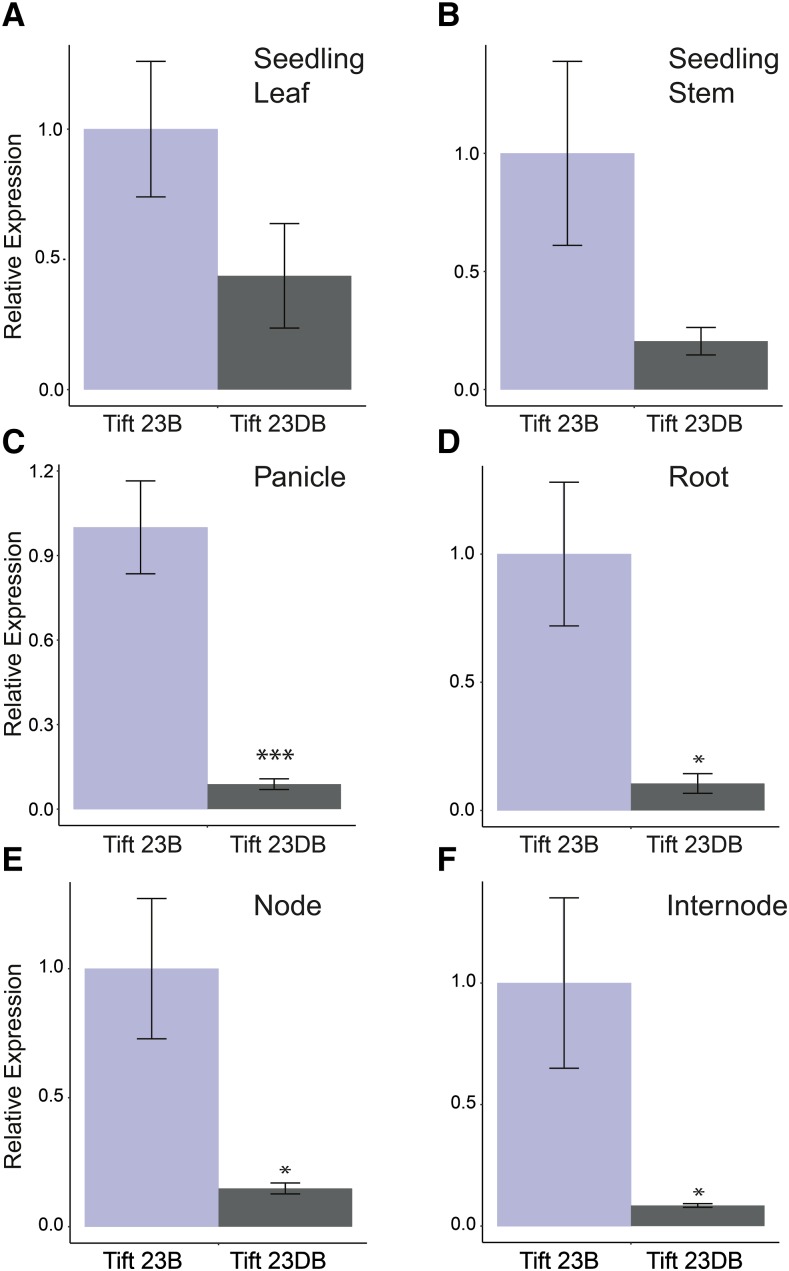
Relative expression of *ABCB1* in Tift 23DB (*d2d2*) and Tift 23B (*D2D2*) in A) leaves at the 5-leaf stage; B) stems at the 5-leaf stage; C) panicles at 50% stigma emergence; D) roots at 50% stigma emergence; E) top node at 50% stigma emergence; and F) top internode at 50% stigma emergence. Expression was normalized using pearl millet *ACTIN* and *GAPDH* (n = 3) expression levels.

Primer efficiencies for the *ABCB1*, *ACTIN* and *GAPDH* genes were highly similar with ranges across the replicates of 84–85% for primer set Ca_ABCB1F20/R20, 84–85% for the *ACTIN* primer set and 83–88% for the *GAPDH* primer set. Expression levels of *ABCB1* were higher in the tall line Tift 23B (*D2D2)* compared to the dwarf Tift 23DB (*d2d2*) both in the two organs tested at the 5-leaf stage (leaf, stem) and the four organs tested at 50% stigma emergence (panicle, top node, top internode, roots), although the difference was statistically significant only at the adult plant stage (Figure 3, Table S3). Tift 23B and Tift 23DB are near-isogenic but differ in plant height at the *D2* locus. Plant height of Tift 23B and Tift 23DB was significantly different at 50% stigma emergence (*P* = 0.03). However, no statistically significant differences in height were observed between the tall and *d2* dwarf near-isogenic lines Tift 23B and Tift 23DB at the 5-leaf stage (*P* = 0.27) (Figure S4A). Expression analyses in stems collected at the five-leaf stage from an additional two tall (ICMP 451, P-1449-2) and two dwarf accessions (81B, pT 732B) confirmed that *ABCB1* was expressed significantly higher in a subset of *D2* lines compared to *d2* lines as early as the five-leaf stage (Figure S4B; Table S3).

### Heterologous transformation studies in Arabidopsis

**Figure 4:**
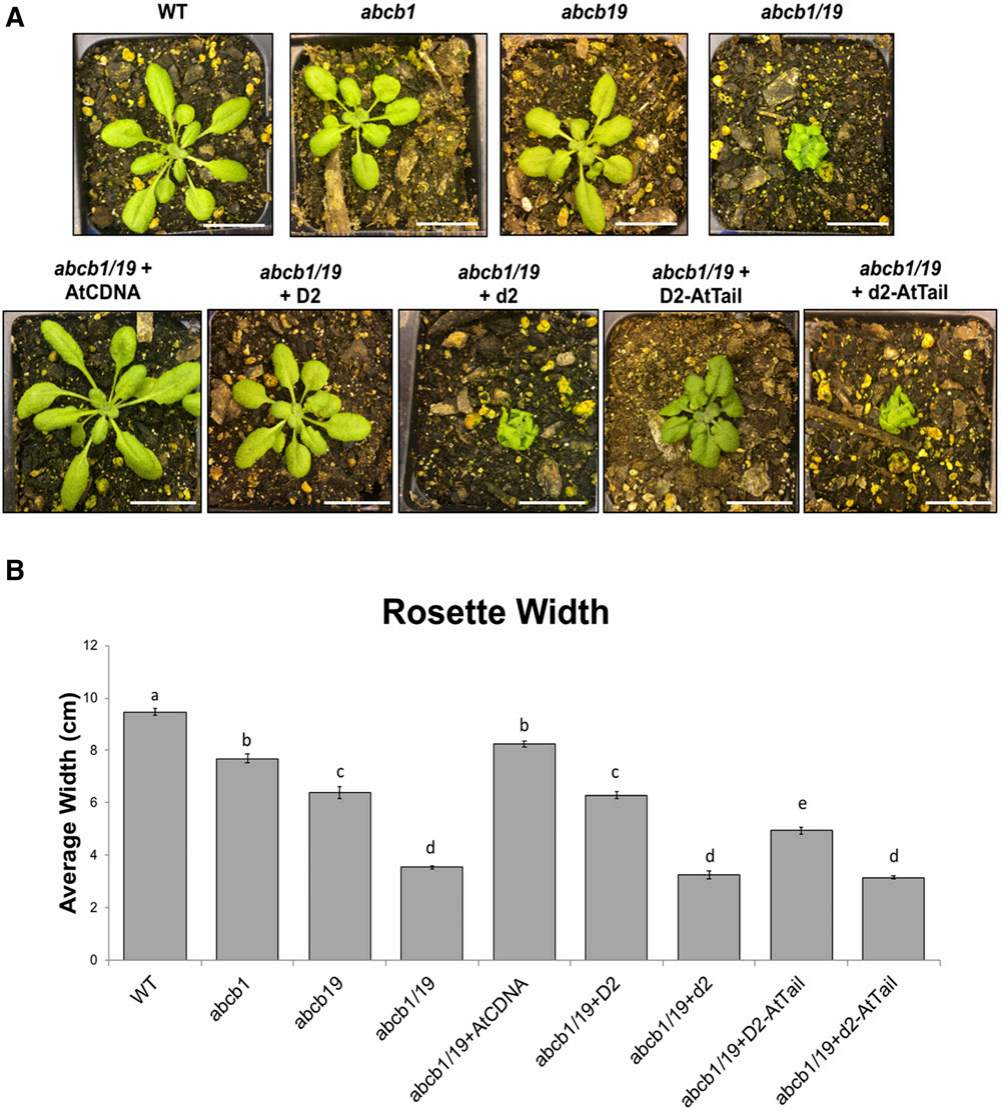
A) Phenotypes and B) rosette width measurments of *abcb1*, *abcb19* and *abcb1abcb19* mutants and of representative plants transformed with At_CDNA, D2, d2, D2_AtTail and d2_AtTail constructs. The scale bar shown in A) represents 1 inch. Statistical significance as indicated by letters in B) was for P-values < 0.0001.

To test whether the ‘Juriah’ insertion was sufficient to inactivate *ABCB1*, we transformed the *Arabidopsis abcb1/abcb19* double mutant with *Arabidopsis ABCB1* (AtCDNA construct; control), pearl millet *ABCB1* (D2 construct; *D2* allele) and pearl millet *abcb1* (d2 construct; similar to *d2* allele but containing a ‘Juriah’ solo LTR instead of a full-length element). Two additional constructs (D2_AtTail and d2_AtTail; pearl millet *ABCB1* and *abcb1*, respectively, with the last 78 bp derived from *Arabidopsis ABCB1*) were generated to test the effect of the tail region of *ABCB1*, which is highly variable between species, on function. Two to 14 plants from at least three independent transgenic lines were grown for each of the five constructs and their phenotype compared with that of wild-type, single mutant *abcb1* and *abcb19*, and double mutant *abcb1abcb19* plants. Functional *ABCB1* alleles were expected to rescue the *abcb1abcb19* phenotype, that is to yield a phenotype similar to that of the *abcb19* single mutant. Plants were grown under short days to intensify the dwarfing phenotype of the *abcb1 Arabidopsis* mutant (Noh *et al.* 2001; Geisler *et al.* 2005). Nevertheless, phenotypes of the single mutants were similar to that of the wild-type for several of the traits measured (Figure S5; Table S4; Table S5). The largest difference between the wild-type and single mutants was observed in the rosette width (Figure 4). The double *abcb1abcb19* mutant had a dwarf phenotype and was significantly different from the wild-type and single mutants for all phenotypic characters measured. Transgenic lines containing the *Arabidopsis* AtCDNA construct rescued the *abcb1abcb19* dwarf phenotype. Rosette width, leaf length and width, inflorescence stem height and flowering time of the AtCDNA overexpression lines were similar or larger than the corresponding traits in the *abcb19* single mutant (Figure 4; Figure S5; Table S3). Similarly, the pearl millet D2 construct rescued *abcb1abcb19* dwarf phenotypes to *abcb19* levels for all traits measured. In contrast, the pearl millet d2 construct which carried a solo LTR in exon 1 of *ABCB1* did not rescue dwarf phenotypes and was morphologically similar to the *abcb1abcb19* double mutant. Semi-quantitative RT-PCR of a subset of the transformants showed that the transgene was expressed in all lines tested, including the d2 and d2_AtTail constructs which had phenotypes similar to that of the double *abcb1abcb19* mutant (Figure S6).

Interestingly, transformants with the D2_AtTail construct, which carried the pearl millet *ABCB1* gene in which the last 78 bp were replaced with the corresponding *Arabidopsis* COOH-terminal region, only partially rescued the *abcb1abcb19* phenotype (Figure 4; Figure S5; Tables S4, S5). As expected, transformants with the d2_AtTail construct showed similar phenotypes to *abcb1abcb19* mutants and transgenic *d2* plants (Figure 4; Figure S5; Tables S4, S5).

## Discussion

### *ABCB1* underlies the d2 phenotype

*ABCB1* was suggested as a candidate for *d2* based on comparative map information (Parvathaneni *et al.* 2013) and this was the impetus for isolation of the full length *ABCB1* alleles from tall (*D2*) and dwarf *(d2*) inbred pearl millet lines. To ensure that we indeed isolated *ABCB1*, a neighbor-joining tree was constructed of the *in-silico* translated *D2* sequence obtained, and the protein sequences of ABCB1 and its closest homolog ABCB19 from *Sorghum bicolor*, *Zea mays*, *Oryza sativa*, *Setaria italica*, *Brachypodium distachyon* and *Arabidopsis thaliana*. The phylogenetic placement of the D2 protein confirmed that the isolated sequence was *Ca_ABCB1* (Figure S3). *ABCB1* orthologs carried variable numbers of introns, ranging from one to nine. The cause of the dynamic structure of *ABCB1* is unknown (Parvathaneni *et al.* 2017).

The *ABCB1* alleles isolated from a tall and dwarf pearl millet inbred differed in their coding region by the presence of a ∼15 kb LTR retrotransposon. A second LTR retrotransposon was present in the presumed promoter region, 664 bp upstream of the *ABCB1* start codon. Since the LTR-retrotransposon insertion in exon 1 disrupts the first transmembrane domain of ABCB1, any ABCB1 protein that is formed in the *d2* dwarf is almost certainly inactive. Based on the homology between the 5′ and 3′ LTR sequences, insertion of the ‘Juriah’ and ‘Parel’ elements was estimated to have occurred in the past 42,000 years and 57,000 years, respectively. Once the LTR retrotransposon insertion inactivated the *ABCB1* gene, presumably *Ca_abcb1* was no longer under any selective constraints and free to accumulate mutations. However, only a single synonymous SNP differentiates the 5.4 kb open reading frame of *Ca_abcb1* and *Ca_ABCB1*, narrowing the time frame of gene inactivation to the past 14,000 to 7000 years. Furthermore, if the observed synonymous SNP was the result of standing variation, the insertion of the LTR retrotransposons may have occurred in more recent times.

RepeatMasker analysis of the ‘Juriah’ and ‘Parel’ TEs against the pearl millet genome sequence (http://gigadb.org/dataset/100192) showed that these elements made up ∼7% and ∼2% of the genome, respectively. According to Estep *et al.* (2013), ‘Juriah’ is the third most abundant LTR retrotransposon in *Cenchrus americanus* covering a total of 86.8 Mb. Typically, high copy LTR retrotransposons insert into one another and are inactivated through methylation (Sanmiguel *et al.* 1998). A number of genes that show loss of function through the insertion of LTR retrotransposons have been reported. Independent insertions of *TSI-7* (gypsy-like) and *TSI-9* (copia-like) elements in exon 3 and exon 9 of the *granule-bound starch synthase 1 (GBSS1)* gene in *Setaria* caused the conversion of non-waxy grain to waxy grain (Kawase *et al.* 2005). The *TSI-7* TE has a higher copy number in domesticated foxtail (*Setaria italica*) than its wild progenitor, green foxtail (*Setaria viridis*) and has been classified as “recently active” (Hirano *et al.* 2011). The terminal repeat retrotransposon in miniature (TRIM) insertion in the coding region of the *cycloidea-like* gene *HaCYC2c* in sunflower led to tubular ray florets (*HaCYC2c-tub* allele) (Chapman *et al.* 2012). The insertion of the *Gret1* LTR retrotransposon in the promoter of the *myb-related transcription factor VvmybA2* gene in grape eliminated *VvmybA2* expression, leading to a loss of red pigmentation (Kobayashi *et al.* 2004; Pereira *et al.* 2005). In most cases, the inserting elements had low to moderate copy numbers. An exception was the potentially active high copy transposon *Rider* which inserts into or near genes in tomato and is thought to have played an important role in tomato evolution and domestication (Jiang *et al.* 2009).

### Origin of the d2 dwarf

In 1966, Burton and Fortson (1966) described five dwarf pearl millet inbreds, two of which carried a single recessive gene for reduced height and received the designations *d1* and *d2*. The exact source of the *d2* dwarf mutation, however, was not reported. We had earlier hypothesized that the *d2* mutation had been present in heterozygous condition in one of five pearl millet accessions that Burton had acquired from the Vavilov Institute of Plant Industry in the mid-1930s (Parvathaneni *et al.* 2013). A leafy dwarf was identified among progeny from a plant obtained through mass selection from those introductions and was subsequently used in crosses with an adapted pearl millet line to generate the synthetic cultivar ‘Starr’. The morphological characteristics of ‘Starr’ fit the description of a *d2* dwarf (Burton and Devane 1951). Seeds of ‘Starr’ millet (NSL 4716) were obtained from the USDA National Plant Germplasm System (NPGS). However, the ‘Starr’ millet obtained was tall and thus likely different from the ‘Starr’ millet described by Burton and Devane (1951). As expected for a tall line, both the ‘Juriah’ and ‘Parel’ LTR-retrotransposons were absent from ‘Starr’ *ABCB1*.

### The expression profile of ABCB1 in pearl millet

Expression of *ABCB1* was observed in all the analyzed organs in pearl millet (leaves and stem at 5-leaf stage; nodes, internodes, panicle and root at 50% stigma emergence). The pearl millet *ABCB1* expression pattern was similar to that observed in *Arabidopsis* where *ABCB1* is expressed at high levels in a range of tissues with the highest expression levels being observed in inflorescence nodes (Titapiwatanakun and Murphy 2009). In maize, *ABCB1* has also been reported to be expressed in a range of tissues. Low expression levels were observed in maize kernels and the root elongation zone (Forestan *et al.* 2012). Although the vascular architecture and development of shoot apical meristems differ between monocots and dicots, the role of *ABCB1* has been shown to be conserved between maize and *Arabidopsis* (Knöller *et al.* 2010). The similar *ABCB1* expression profile in pearl millet suggests functional conservation of *ABCB1* in pearl millet as well.

*ABCB1* transcript levels, which were measured downstream of the ‘Juriah’ element, were significantly lower in *d2* dwarfs compared to tall inbreds (Figure 3; Table S3; Figure S4). Although it is possible that the 15 kb ‘Juriah’ element reduces the rate of transcription, we consider it more likely that the presence of the retrotransposon in exon 1, in addition to disrupting the *ABCB1* coding region, reduces the stability of *ABCB1* transcripts. Destabilization of transcripts caused by the insertion of long interspersed nuclear element (LINE) retrotransposons in introns has been shown in humans (Chen *et al.* 2006). Because the plant promoter prediction program TSSP (Solovyev and Salamov 1997) predicted the presence of one TATA box and one enhancer at the 3′ end of the LTR of the ‘Juriah’ transposon with a linear discriminant function (LDF) weight of 0.11 and 0.09, respectively (threshold LDF for identifying TATA boxes = 0.02; Enhancer = 0.04), we also considered whether the observed *ABCB1* transcripts in the *d2* dwarf were initiated from the ‘Juriah’ 3′ LTR. If this were the case, we would expect transcript levels to be similar across different organs in dwarf lines. The fact that transcript levels in different organs (node, internode, panicle and root at 50% flowering) follow the same trend in dwarf lines as in tall lines (r = 0.91; *P* = 0.095) is congruent with the hypothesis that reduced transcript levels are caused by transcript instability.

### The quest for independent d2 mutations

Identification of independent mutant alleles is one way to confirm the identity of a candidate gene. Several *d2* mutants had previously been recovered from mutagenesis experiments with gamma radiation and salicylic acid (Sukhadev *et al.* 1987), but seed of these *d2* mutants was no longer available (MV Subbarao; personal communication). However, nine *d2* dwarfs were obtained that had been reported as spontaneous and thus, presumably, represented independent mutants (Appa Rao *et al.* 1986; CT Hash, pers. comm.). Analysis of these spontaneous *d2* dwarfs with transposon-gene boundary primers, however, demonstrated that the ‘Juriah’ and ‘Parel’ elements were present in the coding region and promotor region, respectively, of all presumed independent pearl millet *d2* dwarf mutants tested. The ‘spontaneous’ mutants, which were discovered in tall African landraces in the field in India, most likely originated through outcrossing with Indian *d2* germplasm.

### Heterologous transformations of Ca_ABCB1 in Arabidopsis

Because no independent pearl millet *d2* mutants could be identified, and virus-induced gene silencing had not yet been achieved in pearl millet, we aimed to demonstrate that the presence of the ‘Juriah’ element indeed inactivated the pearl millet *ABCB1* gene by transforming *Ca_ABCB1* in an *abcb1* background. However, transformation in pearl millet is not routine and transformation efficiency in cereals is still largely genotype-dependent (Ji *et al.* 2013). We therefore conducted our confirmation studies in *Arabidopsis thaliana*. Single *abcb1* mutants in *Arabidopsis* either have no or a very weak phenotype, likely because of partial functional redundancy between *ABCB1* and its close homolog *ABCB19* (Noh *et al.* 2001; Figure 4). This is in contrast to grasses which, despite carrying two copies of *ABCB19* (Figure S3), display a clear dwarf phenotype upon knockout of *ABCB1*. Double mutants of *abcb1* and *abcb19* display an extreme dwarf phenotype in *Arabidopsis* (Figure 4). We transformed a *de novo* generated *abcb1abcb19* double mutant with five constructs. The AtCDNA construct which carried the *Arabidopsis ABCB1* coding region under control of a 35S promoter served as control. The D2 and d2 constructs carried the pearl millet *ABCB1* coding region under control of a 35S promoter, and differed by the presence of a ‘Juriah’ solo LTR that we engineered into exon 1 of the d2 construct. We reasoned that a ∼1.4 kb solo LTR would be equally efficient in interrupting *ABCB1* function as a ∼15 kb full-length LTR retrotransposon, and would be much easier to manipulate than a full-length element. We also generated two constructs with chimeric pearl millet - *Arabidopsis ABCB1* genes, one for the functional allele (D2_AtTail) and one for the non-functional allele (d2_AtTail) as control, in which the last 78 bps of the pearl millet *ABCB1* coding region were replaced with the 78 bps immediately upstream from the *ABCB1* stop codon in *Arabidopsis*. The carboxy-terminal 20 amino acids are highly variable in the ABCB1 protein (Figure S7). The COOH-terminus of ABCB1 interacts with the TWISTED DWARF1 (TWD1) protein (Geisler *et al.* 2003) and this interaction is vital for ABCB1-mediated transport of auxin (Bailly *et al.* 2014). We hypothesized that, if the variable region was important in the ABCB1-TWD1 interaction, the pearl millet ABCB1 protein might not be able to interact with the *Arabidopsis* TWD1 protein.

Both the AtCDNA and D2 constructs rescued the *abcb1abcb19* dwarf phenotype. As expected, transformants carrying the d2 and d2_AtTail constructs had the same phenotype as the *abcb1abcb19* double mutant, which demonstrated that the insertion of an LTR in exon 1 of *ABCB1* indeed abolished gene function. Interestingly, the chimeric protein (D2_AtTail) had reduced functionality compared to the pearl millet ABCB1 protein. The transgenic *Arabidopsis* lines expressing the D2_AtTail construct had phenotypes that were intermediate between those of the *abcb1abcb19* double mutant and transformants carrying the D2 construct. The intermediate phenotype indicates that the variable COOH-terminal region affects ABCB1 function, but is unlikely to be involved in TWD1 binding. In the mammalian Multidrug Resistance Protein 1 (MRP1), an ABCC1 transporter, removal of as few as four COOH-terminal amino acids decreased nucleotide binding by NBD2 and greatly reduced transport activity (Westlake *et al.* 2004). Westlake and colleagues hypothesized that removal of the four COOH-terminal amino acids destabilized the upstream helix structural motif. Replacement of the COOH-terminal region from MRP1 with comparable regions from the homologous MRP2 (ABCC2) or distantly related P-glycoprotein P (ABCB1), however, did not affect protein activity despite little amino acid identity between the substituted regions (Westlake *et al.* 2004). Similarly to mammalian MRP1, the COOH-terminal region of pearl millet ABCB1 is predicted to have a sheet-sheet-helix-helix structure. The most terminal helix is adjacent to the variable region and has the sequence ‘CYARMLQLQR’ in pearl millet and the chimeric construct, and ‘(I)YARMIQLQR’ in *Arabidopsis* (Figure S7). The isoleucine residue in parenthesis is not predicted to be part of the helix motif in *Arabidopsis*. We hypothesize that, as in mammalian MRP1, the variable COOH-region stabilizes the adjacent helix motif and hence is required for full activity of ABCB1. Although Westlake and colleagues showed that the variable tail region in mammalian ABCC1 could be replaced with that of ABCC2 and ABCB1 proteins, both the C-terminal helix and the tail were replaced in those experiments. In our experiments, the pearl millet helix motif was combined with the *Arabidopsis* variable C-terminus.

## Conclusions

Isolation of the *ABCB1* gene underlying the *d2* dwarf phenotype demonstrated that tall and *d2* dwarf plants differ by the presence of two transposable elements, one in the coding region and one in the presumed promoter. Insertion of the ‘Juriah’ element in exon 1 led to inactivation of the ABCB1 protein. Because no independent mutations in *ABCB1* have been identified, it seems likely that all *d2* mutants grown worldwide originated from a single source plant. While inactivation of ABCB1 led to agronomically useful dwarf phenotypes in pearl millet and sorghum, in maize, the resulting knock-out phenotype was extreme. An amino acid substitution in the second transmembrane domain, however, caused a moderate dwarfing phenotype with potential for maize improvement (Xing *et al.* 2015). We demonstrated that modifying the variable COOH-terminus of ABCB1 also partially reduced protein activity. It would be interesting to test whether a COOH-modified ABCB1 would represent a novel semi-dwarfing phenotype in monocots.
